# Comparative study on the spectral properties of boron clusters B_n_^0/−1^(n = 38–40)

**DOI:** 10.1038/srep25020

**Published:** 2016-04-26

**Authors:** Shixiong Li, Zhengping Zhang, Zhengwen Long, Guangyu Sun, Shuijie Qin

**Affiliations:** 1 College of Big Data and Information Engineering, Guizhou University, Guiyang 550025, China; 2 School of Physics and Electronic Science, Guizhou Education University, Guiyang 550018, China; 3 College of Science, Guizhou University, Guiyang 550025, China; 4 Key Lab of Photoelectron Technology and Application, Guizhou University, Guiyang 550025, China

## Abstract

The all-boron fullerenes B_40_^−1^ and B_39_^−1^ discovered in recent experiments are characterized and revealed using photoelectron spectroscopy. Except for the photoelectron spectroscopy, one may identify such boron clusters with other spectroscopic techniques, such as infrared spectra and Raman spectra. Insight into the spectral properties of boron clusters is important to understand the boron clusters and find their potential applications. In this work, density functional theory (DFT) and time-dependent density functional theory (TD-DFT) calculations are carried out to comparatively study the vibrational frequencies, infrared spectra, Raman spectra and electronic absorption spectra of boron clusters B_n_^0/−1^(n = 38–40). The numerical simulations show that such boron clusters have different and meaningful spectral features. These spectral features are readily compared with future spectroscopy measurements and can be used as fingerprints to distinguish the boron clusters B_n_^0/−1^ with different structures (cage structure or quasi-planar structure) and with different sizes (n = 38–40).

Discovery of C_60_ has enriched the chemistry of carbon and leaded to new carbon-based nanomaterials^1^,^2^,^3^,^4^. However, similar boron fullerenes have not been received enough attention since boron is an electron deficient atom with only three valence electrons. Over the past decade, experimental and theoretical efforts have been used to systematically elucidate the electronic and structural evolution of boron clusters. And previous works have show that most boron nanoclusters are planar or quasi-planar structures^5^^–^12. An intriguing fullerene-like cluster B_80_, which has the same valance electrons with C_60_, was predicted in 2007^13^. Subsequently, fullerene-like B_80_ was found not to be the global minimum, and the most favorable B_80_ is likely a core-shell type three-dimensional structure^14^,^15^. Since the first proposal of a possible B_80_ cage, the pursuit of boron cages has attracted significant computational activity in the past several years^16^,^17^,^18^,^19^,^20^. Nevertheless, seeking for all-boron clusters with fullerene-like structure is still a challenge due to the geometrical frustration arising from competitions among various structural motifs. Lv *et al.*^21^ reported a B_38_ fullerene analogue with high symmetry (D_2h_) and consists of 56 triangles and four hexagons. In spite of B_38_ with fullerene-like structure is the global minimum of the cluster B_38_ and much more stable than the quasi-planar structure, recent theoretical study has show that both fullerene-like structure and quasi-planar B_38_ can be considered to be of a transition size between 2D and 3D boron clusters^22^. The quasi-planar B_38_ is more stable than fullerene-like B_38_ based on the results computed using CCSD method.

There has been no experimental evidence of the existence of all-boron fullerene in the past several decades. Recently, an all-boron fullerene-like cage cluster B_40_^−^ was produced in a laser vaporization supersonic source^23^. Both B_40_^−^ and the neutral counterpart B_40_ exhibit the fullerene-like cage containing two hexagonal and four heptagonal holes, relevant theoretical simulations indicated that neutral cage cluster B_40_ is the most stable structure among the isomers of B_40_. The first all-boron fullerene B_40_ is named ‘borospherene’. Soon after, the cage cluster B_39_^−^ was also produced via laser vaporization^24^. Experimental and theoretical studies shown that B_39_^−^ has a C_3_ cage global minimum with a low-lying C_2_ cage isomer, both the C_3_ and C_2_ B_39_^−^ cages are chiral with degenerate enantiomers, the anionic B_39_^−1^ has a closed-shell electronic structure and neutral B_39_ is thus a superhalogen species. These experiment studies also arouse interest in boron fullerenes and boron-based nanomaterials, such as dynamical behavior of B_40_ fullerence^25^, hydrogen storage capacity of Ti-decorated B_40_ fullerence^26^, structures and electronic properties of endohedral derivatives M@B_40_(M = Na, Sc, Y, La, Ca, Sr), exohedral derivatives M&B_40_(M = Na, Be, Mg)^27^,^28^,^29^ and optical spectra of neutral B_40_ clusters^30^. As the discovery of C_60_, the observation of the all-boron fullerene will lead to a new beginning for the study of boron fullerenes, both experiment and theory, which may lead to new boron-based nanomaterials.

Optical properties of nanoclusters have dependency on size and structure^31^, due to the quantum confinement effect, size and structure of materials can influence the energy gap between highest occupied orbital (HOMO) and lowest unoccupied molecular orbital (LUMO). It is necessary to study the spectral characteristics of medium-sized boron clusters, especially the boron fullerenes, current work is therefore to provide a comparative theoretical study on the infrared, Raman and electronic absorption spectra of boron clusters B_n_^0/−1^ (n = 38–40) based on the DFT method and TD-DFT method. Although the spectra of neutral B_40_ were reported^30^, we are unaware of such a study on other boron clusters, especially a detailed theoretical study. Our current study can provide valuable results to assist further experimental measurements on these boron clusters and derivatives, and also may provide theoretical guidance for the application of these boron clusters in the future.

All ground-state geometries and frequency calculations of these boron clusters are performed based on the density functional method PBE0 with 6–311 + G^*^ basis set. These optimized structures are used in the calculations of electronic absorption spectra based on the time-dependent DFT formalism at the same level. The method used in our work has been used in previous papers^23^,^24^,^32^, it is reliable for boron clusters. In the previous papers, ground state geometries and relevant calculations of B_n_^0/−1^(n = 38–40) were performed using different methods with different basis sets. The initial structures of B_n_^0/−1^(n = 38–40) in our work are derived from the corresponding papers^21^,^23^,^24^. Although ground state geometries of the B_40_ and B_39_^−1^ were optimized using density functional method PBE0 with the basis set 6–311 + G*, to obtain the relative comparison, all ground state geometries of B_n_^0/−1^(n = 38–40) are also re-optimized using the same method. All computations are carried out with the Gaussian09 software package^33^.

## Results and Discussion

Optimized structures of boron clusters B_n_^0/−1^(n = 38–40) are depicted in Fig. S1 (Supplementary Information). Both B_40_ and B_40_^−1^ cages contain two hexagonal and four heptagonal rings, B_38_ and B_38_^−1^ cages contain four hexagonal rings. Cage clusters B_39_^0/−1^ with C_3_ structure contain three hexagonal and three heptagonal rings, however, cage clusters B_39_^0/−1^ with C_2_ structure contain two hexagonal and four heptagonal rings. Ground state parameters are summarized in Table 1, which are consistent with the previous literature^21^,^23^,^24^. As given in Table 1, B_40_ cage has the largest HOMO–LUMO energy gap of 3.13 eV among all boron clusters predicted here and it is larger than 3.01 eV of C_60_. In general, HOMO–LUMO energy gap reflects the ability for an electron to jump from the occupied orbital to the unoccupied orbital, which represents the intensity of chemical activity. A large HOMO–LUMO gap generally corresponds to a closed-shell electronic configuration with high stability. For the cage clusters with closed-shell electronic structure, B_40_ has the largest energy gap, which indicates that its chemical activity is lowest. It is noticeable that energy gaps reduce from 3.13 eV of B_40_ cage to 2.23 eV of B_38_ cage, which verifies that the size of cage cluster has a great influence on the HOMO–LUMO energy gap. In addition, dipole moments of cage cluster B_38_^0/−1^ and B_40_^0/−1^ are zero among all the boron clusters because of the highly symmetric structures (D_2h_ and D_2d_), this indicates that they do not render far-infrared pure rotation spectrum.

**Figure 1:**
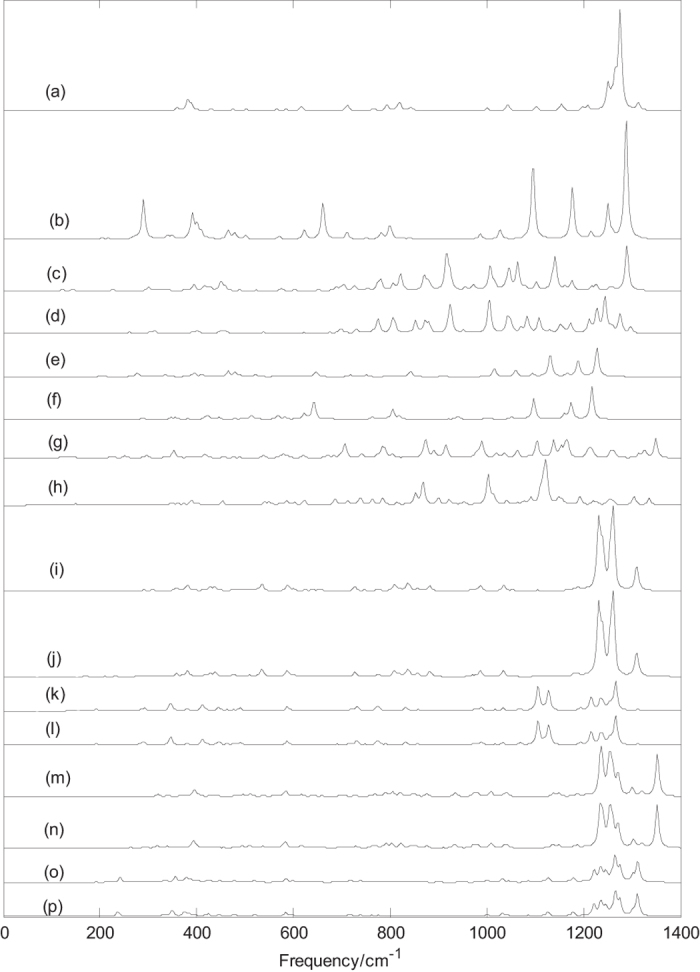
Predicted infrared spectra of boron clusters B_n_^0/−1^(n = 38–40) based on PBE0 functional with 6–311 + G^*^ basis set. (**a**) D_2d_ B_40_, (**b**) D_2d_ B_40_^−1^, (**c**) C_s_ B_40_, (**d**) C_s_ B_40_^−1^, (**e**) D_2h_ B_38_, (**f**) D_2h_ B_38_^−1^, (**g**) C_1_ B_38_, (**h**) C_1_ B_38_^−1^, (**i**) C_3_ (1) B_39_^−1^, (**j**) C_3_ (2) B_39_^−1^, (**k**) C_3_ (1) B_39_, (**l**) C_3_ (2) B_39_, (**m**) C_2_ (1) B_39_^−1^, (**n**) C_2_ (2) B_39_^−1^, (**o**) C_2_ (1) B_39_ and **(p**) C_2_ (2) B_39_.

**Figure 2:**
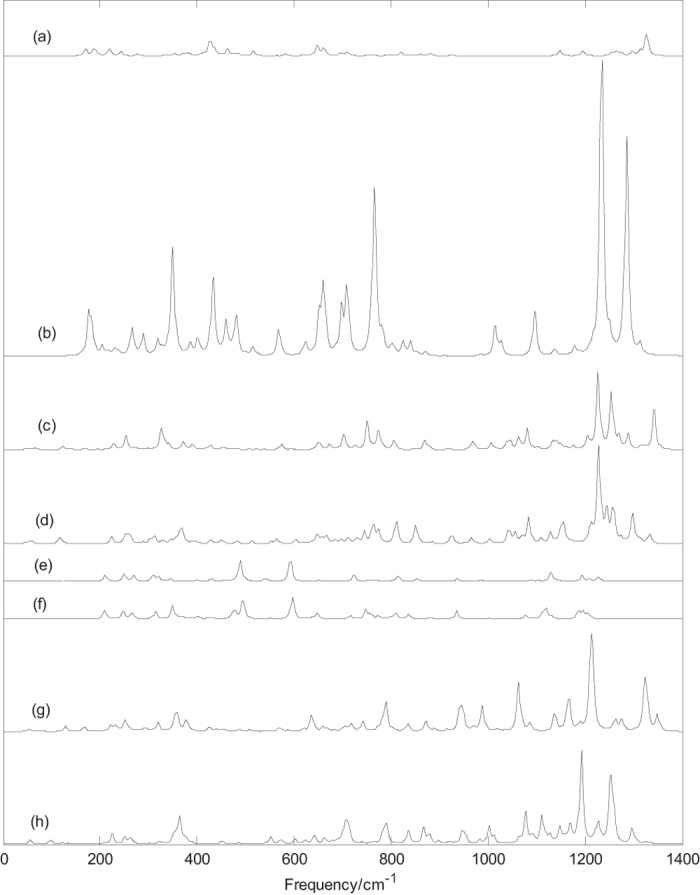
Predicted Raman spectra of boron clusters B_40_^0/−1^ and B_38_^0/−1^ based on PBE0 functional with 6–311 + G^*^ basis set. (**a**) D_2d_ B_40_, (**b**) D_2d_ B_40_^−1^, (**c**) C_s_ B_40_, (**d**) C_s_ B_40_^−1^, (**e**) D_2h_ B_38_, (**f**) D_2h_ B_38_^−1^, (**g**) C_1_ B_38_ and (**h**) C_1_ B_38_^−1^.

**Figure 3:**
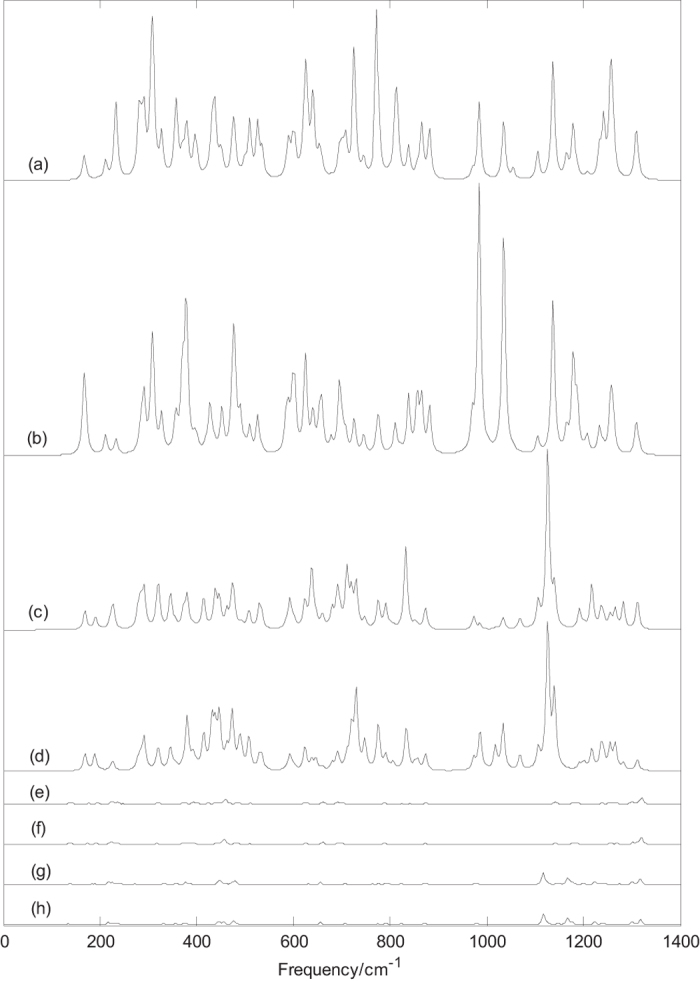
Predicted Raman spectra of boron clusters B_39_^0/−1^ based on PBE0 functional with 6–311 + G^*^ basis set. (**a**) C_3_ (1) B_39_^−1^, (**b**) C_3_ (2) B_39_^−1^, (**c**) C_3_ (1) B_39_, (**d**) C_3_ (2) B_39_, (**e**) C_2_ (1) B_39_^−1^, (**f**) C_2_ (2) B_39_^−1^, (**g**) C_2_ (1) B_39_ and (**h**) C_2_ (2) B_39_.

Normal mode frequencies, infrared intensities and Raman activities of B_n_^0/−1^(n = 38–40) are calculated and depicted in Figs 1, 2, 3. Frequency calculations confirm the dynamical stability of these boron clusters by showing no imaginary frequencies. The axially chiral cage clusters B_39_^−1^ with C_3_ symmetry have the same vibrational frequencies as well as axially chiral cage clusters B_39_ with C_3_ symmetry. The lowest vibrational frequency of each cage cluster is 170 cm^−1^ for D_2d_ B_40_, 176 cm^−1^ for D_2d_ B_40_^−1^, 168 cm^−1^ for C_3_ B_39_^−1^, 136 cm^−1^ for C_2_ B_39_^−1^, 170 cm^−1^ for C_3_ B_39_, 135 cm^−1^ for C_2_ B_39_, 205 cm^−1^ for B_38_ and 174 cm^−1^ for B_38_^−1^. All lowest vibrational frequencies are above the stability threshold^34^ of 100 cm^−1^. The lowest frequencies of quasi-planar clusters B_40_, B_40_^−1^, B_38_ and B_38_^−1^ are 49 cm^−1^, 46 cm^−1^, 51 cm^−1^ and 55 cm^−1^, respectively. The highest vibrational frequency of these clusters is lower than 1400 cm^−1^.

Infrared spectra of boron clusters B_n_^0/−1^(n = 38–40) are given in Fig. 1, these infrared spectra peaks distribute in three regions: low frequency region (from 40 cm^−1^ to 600 cm^−1^), middle frequency region(from 600 cm^−1^ to 1000 cm^−1^) and high frequency region (from 1000 cm^−1^ to 1400 cm^−1^), the main characteristic peaks are located in the middle and high frequency regions (from 600 cm^−1^ to 1400 cm^−1^). Vibrational modes of these main peaks contain the stretching and bending vibration of boron atoms. These vibrational modes within the middle and high frequency regions are closely related to the molecular structure. This suggests that molecular with slightly difference can lead to the subtle differences of infrared absorption in these regions, namely, the infrared spectra of molecular show the characteristics of molecular, like fingerprints, known as the fingerprint region.

Figure 1(a) presents the infrared spectra of B_40_ cage, the major peaks appear at 382, 616, 712, 794, 822, 1103, 1153, 1252, 1264, 1274 and 1313 cm^−1^. The sharpest peak occurs at 1274 cm^−1^, this vibrational mode is doubly degenerate vibrational mode and formed by stretching vibration of boron atoms mainly located in the hexagonal rings. Figure 1(b) presents the infrared spectra of B_40_^−1^ cage, the main peaks appear at 289, 392, 402, 622, 662, 799, 1096, 1176, 1252 and 1287 cm^−1^. The sharpest peak occurs at 1287 cm^−1^, this vibrational mode is doubly degenerate and Raman active mode. Unlike the neutral B_40_ cage, this vibrational mode is formed by stretching vibration of boron atoms mainly located in the heptagonal rings. As shown in Fig. 1(a,b) and Table 1, the addition of an electron does not change the symmetry and dipole moment, but lead to two other strong peaks (at 1096 cm^−1^ and 1176 cm^−1^) in the high frequency region and two other strong peaks (at 289 cm^−1^ and 662 cm^−1^), which will be useful to identify the anionic B_40_^−1^ cage and neutral B_40_ cage. The infrared spectra of the B_40_^−1^ cage are quite similar to endohedral derivative Na@B_40_ and exohedral derivatives Na&B_40_^29^, the metal dopant Na in the B_40_ cage changes the IR absorption peaks of B_40_, enhancing some peaks. As the analysis of M@B_40_ (M = Ca, Sr) and M&B_40_ (M = Be, Mg)^27^, metalloborospherenes (Na@B_40_ and Na&B_40_) are characterized as charge-transfer complexes (M^+^ B_40_^−^), where an metal atom donates one electron to the B_40_ cage, resulting in similar features with anionic B_40_^−1^. This indicates that the addition of an electron plays an important role in vibrational modes and infrared intensities. This also means that infrared spectra of anionic clusters B_n_^−1^(n = 38–40) have the potential for the comparative analysis of metal-doped derivatives (M^1+^B_n_^1^^−^) in future experimental and theoretical researchs. In addition, at 289 cm^−1^, the characteristic peak of B_40_^−1^ is strong, which is different from all other boron clusters. This strong peak is produced by bending vibration of boron atoms and it belongs to the far-infrared spectrum. Figure 1(c) presents the infrared spectra of quasi-planar B_40_, the main peaks appear at 396, 449, 781, 823, 870, 917, 1006, 1046, 1064, 1140, 1176, and 1289 cm^−1^. The characteristic peaks of quasi-planar B_40_ are consistent with the previous literature^30^,^32^. The sharpest peak occurs at 1289 cm^−1^, this vibrational mode is Raman active mode and formed by stretching vibration of boron atoms mainly located in the edge of the quasi-planar molecular. Figure 1(d) presents the infrared spectra of the quasi-planar B_40_^−1^, the main peaks appear at 775, 806, 851, 925, 1004, 1042, 1084, 1109, 1228, 1245 and 1275 cm^−1^. The sharpest peak occurs at 1004 cm^−1^, this vibrational mode is Raman active and formed by stretching vibration of boron atoms. Except for the main peaks at 1245, 1289 and 1140 cm^−1^, the infrared spectra of quasi-planar B_40_ and B_40_^−1^ are quite similar. At 1140 and 1289 cm^−1^, the peaks of B_40_ are strong, however, at 1245 cm^−1^, the peak of B_40_^−1^ is strong. These features can be used to distinguish the quasi-planar B_40_ and B_40_^−1^.

Figure 1(e) presents the infrared spectra of D_2h_ B_38_, the main peaks appear at 277, 397, 478, 646, 842, 1015, 1059, 1130, 1189 and 1228 cm^−1^. Figure 1(f) presents the infrared spectra of D_2h_ B_38_^−1^, the main peaks appear at 514, 567, 623, 642, 806, 820, 1098, 1159, 1174 and 1218 cm^−1^. The sharpest peak occurs at 1228 cm^−1^ for D_2h_ B_38_ and 1218 cm^−1^ for D_2h_ B_38_^−1^, the two vibrational modes are Raman inactive modes and formed by stretching vibration of boron atoms mainly located in the hexagonal rings. Figure 1(e,f) show that three strong peaks with similar characteristics are located in high frequency region, but the addition of an electron shifts the three peaks from 1228, 1189 and 1130 cm^−1^ for B_38_ to 1218, 1174, and 1098 cm^−1^ for B_38_^−1^, respectively. In addition, the vibrational mode at 1015 cm^−1^ is strong in D_2h_ B_38_, but D_2h_ B_38_^−1^ dose not exhibit vibrational mode, the situation at 806 cm^−1^ is just the opposite. Figure 1(g) presents the infrared spectra of the quasi-planar B_38_, the main peaks appear at 705, 784, 873, 988, 1105, 1137, 1153, 1162, 1166 and 1350 cm^−1^. The sharpest peak occurs at 1350 cm^−1^, this vibrational mode is Raman active mode and formed by stretching vibration of boron atoms mainly located in the edge of the quasi-planar molecular. Figure 1(h) presents the infrared spectra of the quasi-planar B_38_^−1^, the main peaks appear at 688, 851, 867, 900, 1002, 1013, 1120, 1192, 1304, and 1336 cm^−1^. The sharpest peak occurs at 1120 cm^−1^. The quasi-planar B_38_^−1^ has three strong characteristic peaks at 1120, 1002 and 867 cm^−1^, other peaks are relatively weak. However, the main peaks of quasi-planar B_38_ show the nearly same intensities. The addition of an electron weakens some strong vibrational modes and leads to three strong characteristic peaks.

Figure 1(i,j) indicate that the two enantiomers C_3_ B_39_^−1^ have the same infrared spectra, the main peaks appear at 382, 536, 586, 726, 837, 985, 1232, 1237, 1241, 1256, 1261 and 1309 cm^−1^. The sharpest peak occurs at 1261 cm^−1^, this vibrational mode is formed by stretching vibration of boron atoms. Figure 1(k,l) indicate that the two enantiomers C_3_ B_39_ also have the same infrared spectra, the main peaks appear at 346, 586, 730, 773, 1107, 1126, 1216, 1236, 1241 and 1265 cm^−1^, and sharpest peak occurs at 1265 cm^−1^. It’s worth noting that neutral B_39_ cage and anionic B_39_^−1^ cage with C_3_ symmetry can be identified through the characteristic peaks at 1107 cm^−1^, 1126 cm^−1^, and 1309 cm^−1^. Figure 1(m,n) show that the two enantiomers C_2_ B_39_^−1^ have the almost same infrared spectra. The sharpest peak occurs at 1353 cm^−1^ for C_2_ (1) B_39_^−1^ and 1352 cm^−1^ for C_2_ (2) B_39_^−1^, the two vibrational modes are formed by stretching vibration of a boron atom located in the adjacent heptagonal rings. Figure 1(o,p) show that the two enantiomers C_2_ B_39_ have the same infrared spectra, the sharpest peak occurs at 1264 cm^−1^. It’s worth noting that neutral B_39_ cage and anionic B_39_^−1^ cage with C_2_ symmetry can be identified through the characteristic peaks at 1311 cm^−1^ and 1352 cm^−1^. Figure 1(i–p) show that the main characteristic peaks of B_39_^0/−1^ distribute in high-frequency region (from 1000 to 1400 cm^−1^), and other peaks are relatively weak. The addition of an electron enhance or weaken these characteristic peaks, lead to significative infrared spectra, such notable differences in infrared spectra of B_39_^0/−1^ can be used as the fingerprint of their existence.

As mentioned before, the sharpest peak of each boron cluster is formed by stretching vibration of boron atoms. One can also observe that the main strong peaks of these boron clusters almost in the mid-infrared region. Except for the relatively strong main peaks mentioned here, these boron clusters have many different relatively weak characteristic peaks. The predicted infrared spectra in Fig. 1 show that boron clusters have different spectral features and characteristic peaks, the predicted infrared spectra provide some information in the future experimental characterization. If the infrared spectra of boron clusters are obtained in experiments, these different characteristic peaks can be used as a basis for the identification of these boron clusters. Due to the wide wavelength range of spectrograph means low wavelength resolution, the predicted frequency regions provide a theoretical basis for the selection of the spectrograph in the future experiments. As mentioned before, the main characteristic peaks are located in high frequency region (from 800 cm^−1^ to 1400 cm^−1^), especially, the characteristic peaks of B_39_^0/−1^ are located in high frequency (from 1000 cm^−1^ to 1400 cm^−1^). This indicates that we should concentrate on the high frequency region in experiments to identify these boron clusters and the wavelength range of spectrograph can be further reduced, it will improve the spectral measurement precision. The vibrational modes with lower intensity is difficult to be obtained in experiments, the calculated results may provide an effective data in the vibrational frequency analysis. The calculated results may be used for the analysis of one component of a mixture (for example, boron isomers) combined with other spectral analysis technology.

Figure 2 depicts the Raman spectra of the B_40_^0/−1^ and B_38_^0/−1^, the characteristic peaks of B_40_ are consistent with the previous literature^30^,^32^. Figure 2(a) presents the Raman spectra of the D_2d_ B_40_, the main peaks appear at 170, 188, 427, 463, 517, 648, 662, 1148, 1194, 1264, 1298 and 1326 cm^−1^. The sharpest peak occurs at 1326 cm^−1^, this vibrational mode is infrared inactive mode and formed by stretching vibration of boron atoms located in the heptagonal rings. Among the Raman active modes, two vibrations at 170 cm^−1^ and 427 cm^−1^ belong to typical radial breathing modes. The breathing modes are used to identity the hollow structures in nanotubes. Figure 2(b) depicts the Raman spectra of D_2d_ B_40_^−1^, the main peaks appear at 176, 351, 433, 459, 482, 651, 662, 698, 710, 765, 1015, 1234 and 1286 cm^−1^. The sharpest peak occurs at 1234 cm^−1^, like the D_2d_ B_40_, this vibrational mode is infrared inactive mode and formed by stretching vibration of boron atoms mainly located in the heptagonal rings. Similar to D_2d_ B_40_ cage, among the Raman active modes, the two vibrations at 176 cm^−1^ and 433 cm^−1^ belong to typical radial breathing modes. In addition, the two vibrations at 183 cm^−1^ and 459 cm^−1^ can be viewed as breathing modes. Similar to infrared spectra of D_2d_ B_40_^−1^, there are four main Raman peaks in the high frequency region. In addition, Fig. 2(b) indicates that other main Raman peaks are located in the middle and lower frequency regions. It’s worth noting that the Raman spectra of B_40_^−1^ cage are far stronger than that of B_40_ cage and the sharpest peak of B_40_^−1^ cage is stronger than that of B_40_ and B_38_^0/−1^. Figure 2(c) presents the Raman spectra of the quasi-planar B_40_, the main peaks appear at 254, 326, 702, 751, 774, 969, 1064, 1081, 1206, 1227, 1254, 1270, 1289 and 1343 cm^−1^. The sharpest peak occurs at 1227 cm^−1^, this vibrational mode is infrared active mode and mainly formed by stretching vibration of the two boron atoms connecting the two centric hexagonal rings. There are three major peaks in the high frequency region. Figure 2(d) presents the Raman spectra of the quasi-planar B_40_^−1^ (C_s_), the main peaks appear at 252, 312, 370, 667, 763, 811, 851, 1042, 1084, 1157, 1212, 1228, 1258 and 1299 cm^−1^. The sharpest peak occurs at 1228 cm^−1^, this vibrational mode is infrared active and is formed by stretching vibration of boron atoms. Except for the main peaks at 1254 and 1343 cm^−1^, the Raman spectra of quasi-planar B_40_ and B_40_^−1^ are basically similar.

Figure 2(e,f) indicate that D_2h_ B_38_ and B_38_^−1^ have the quite similar Raman spectra. It’s worth noting that the highest intensity peaks of D_2h_ B_38_ and B_38_^−1^ are located in middle frequency region. The sharpest peak occurs at 592 cm^−1^ for D_2h_ B_38_ and 597 cm^−1^ for B_38_^−1^, the two vibrational modes are infrared inactive modes and formed by bending vibration of boron atoms, which can be viewed as breathing mode. Among the Raman active modes of D_2h_ B_38_, the vibration at 490 cm^−1^ belongs to typical radial breathing mode. Similar to the D_2h_ B_38_, among the Raman active modes of D_2h_ B_38_^−1^, the vibration at 495 cm^−1^ belongs to typical radial breathing mode. Figure 2(g) depicts the Raman spectra of the quasi-planar B_38_. The main peaks appear at 636, 789, 947, 988, 1064, 1166, 1213, 1322, 1326 and 1350 cm^−1^. The sharpest peak occurs at 1213 cm^−1^, this vibrational mode is infrared active mode and formed by stretching vibration of boron atoms. Two secondary peaks are located on both sides of the sharpest peak. Figure 2(h) presents the Raman spectra of the quasi-planar B_38_^−1^, the main peaks appear at 365, 791, 867, 1002, 1077, 1112, 1168, 1192, 1229 and 1252 cm^−1^, the sharpest peak occurs at 1192 cm^−1^. A visible difference in the Raman spectra of quasi-planar B_38_ and B_38_^−1^ is the first two sharpest peaks from 1213 and 1326 cm^−1^ for B_38_ to 1192 and 1252 cm^−1^ for B_38_^−1^, respectively. As mentioned before, some vibrational modes of cage-like B_38_^0/−1^ and B_40_^0/−1^ are either infrared inactive or Raman inactive and some vibrational modes are infrared inactive and Raman inactive. As given in Table 1, the dipole moments of cage clusters B_38_^0/−1^ and B_40_^0/−1^ are zero and these clusters are highly symmetric structures, which may lead to the infrared inactive and Raman inactive vibrational modes. The calculated results indicate that all vibrational modes of quasi-planar B_38_^0/−1^ and B_40_^0/−1^ are infrared active and Raman active.

Figure 3 depicts the Raman spectra of B_39_^0/−1^. An interesting phenomenon is that the Raman spectra of B_39_^0/−1^ with C_3_ symmetry are far stronger than that of B_39_^0/−1^ with C_2_ symmetry. Figure 3(a,b) present the Raman spectra of the axially chiral B_39_^−1^ with C_3_ symmetry, unlike the infrared spectra of axially chiral C_3_ B_39_^−1^, the two enantiomers have the different Raman spectra features. Figure 3(a) presents the Raman spectra of the C_3_ (1) B_39_^−1^, the main peaks appear at 231, 280, 307, 431, 526, 625, 726, 773, 813, 865, 985, 1137, 1256 and 1309 cm^−1^. The sharpest peak occurs at 773 cm^−1^, this vibrational mode is located in the middle frequency region. Figure 3(b) presents the Raman spectra of the C_3_ (2) B_39_^−1^, the main peaks appear at 168, 307, 379, 477, 601, 624, 695, 855, 985, 1034, 1137, 1180 and 1261 cm^−1^. The sharpest peak occurs at 985 cm^−1^. Among the Raman active modes of C_3_ B_39_^−1^, the position of typical radial breathing mode is 447 cm^−1^ for C_3_ (1) B_39_^−1^ and 448 cm^−1^ for C_3_ (2) B_39_^−1^. Figure 3(c,d) indicate that the axially chiral B_39_ cages with C_3_ symmetry have the similar Raman spectra instead of same Raman spectra. The sharpest peak occurs at 1126 cm^−1^ for C_3_ (1) B_39_ and 1140 cm^−1^ for C_3_ (2) B_39_. Among the Raman active modes of C_3_ B_39_, the vibration at 446 cm^−1^ belongs to typical radial breathing mode. Figure 3(e,f) depict the Raman spectra of the axially chiral C_2_ B_39_^−1^. Like the infrared spectra of C_2_ B_39_^−1^, the two enantiomers have the almost same Raman spectra. The sharpest peak occurs at 1320 cm^−1^. Among the Raman active modes, the vibrations at 459 cm^−1^ for C_2_ (1) B_39_^−1^ and 456 cm^−1^ for C_2_ (2) B_39_^−1^ can be viewed as breathing mode. Figure 3(g,h) indicate that the two enantiomers C_2_ B_39_ have the almost same Raman spectra, the sharpest peak occurs at 1117 cm^−1^. Among the Raman active modes, the vibrations at 446 cm^−1^ for C_2_ (1) B_39_ and 444 cm^−1^ for C_2_ (2) B_39_ can be viewed as breathing mode. The calculated results indicate that all vibrational modes of B_39_^0/−1^ are infrared active and Raman active.

It is worth noting that the position of radial breathing mode depends on the size of cage boron cluster, as mentioned before, the vibrational frequency of the radial breathing mode of B_40_, B_40_^−1^, C_3_ B_39_, C_3_ B_39_^−1^, B_38_, and B_38_^−1^ are 427 cm^−1^, 433 cm^−1^, 446 cm^−1^, 447 cm^−1^, 490 cm^−1^, and 495 cm^−1^, respectively. The presented data indicate a same relationship with fullerenes^35^: for small fullerenes, its vibrational frequency is relatively large, for larger fullerenes, its value is small. Dependence of frequency of radial breathing mode on the number of atoms in a fullerene is very interesting finding, which can be compared in nature to very well-known relationship between the diameter of a carbon nanotube and the location of its breathing mode in Raman spectra. As the discovery of carbon nanotube, Raman spectra of cage boron clusters can be useful for the analysis of boron nanotube in future studies.

The predicted Raman spectra in Figs 2 and 3 provide some information for future experimental characterization. Raman spectra, as the supplement of infrared spectra, can also be used for the basis of identification of boron clusters. From the infrared and Raman spectra of each boron cluster, we can find, at some frequencies, the boron cluster has strong infrared absorption, but the Raman peaks is very weak (or Raman inactive). However, at some frequencies, the relation is just the opposite. In addition, at some frequencies, both the infrared and Raman peaks are strong. A vibrational mode of molecular with no change of dipole moment is infrared inactive, we can’t obtain the normal mode frequency from the infrared spectral data in experiments. However, this vibrational mode may lead to the change of polarizability, this indicates that the vibrational mode is Raman active. The calculated Raman spectra can be useful for analytical purposes and contribute significantly to spectral interpretation and vibrational assignments, also can provide technical guidance for future experiment measurement.

**Figure 4:**
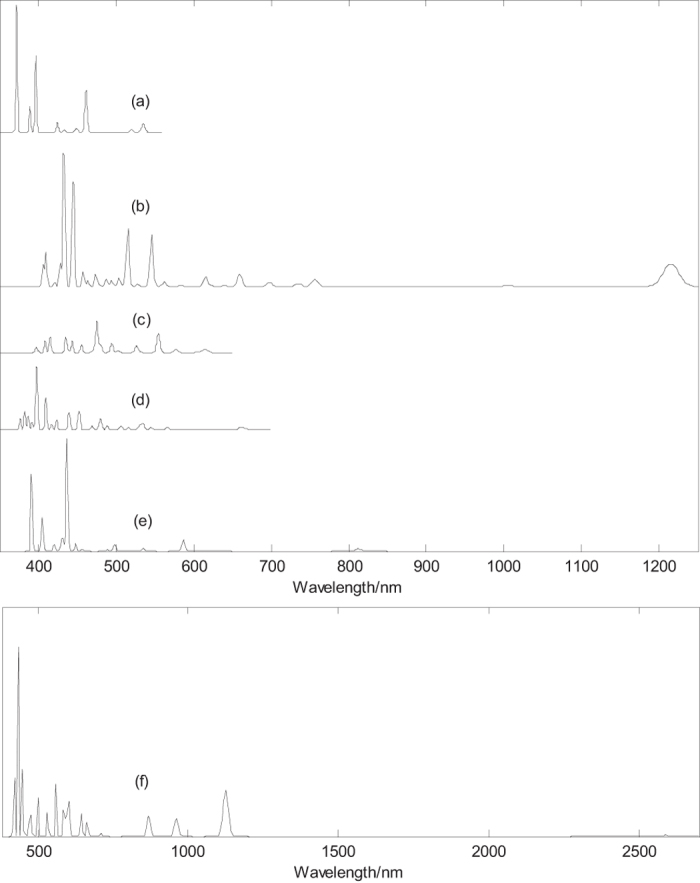
Predicted electronic absorption spectra of boron clusters B_n_^0/−1^ (n = 38–40) with closed-shell electronic structure based on PBE0 functional with 6–311 + G* basis set. (**a**) D_2d_ B_40_, (**b**) C_s_ B_40_, (**c**) C_3_ (1&2) B_39_^−1^, (**d**) C_2_ (1&2) B_39_^−1^, (**e**) D_2h_ B_38_ and (**f**) C_1_ B_38_.

To provide some information for future experimental characterization, we have calculated electronic absorption spectra (the first 36 exited states) of boron clusters B_n_^0/−1^ (n = 38–40) with closed-shell electronic structure, as shown in Fig. 4. Figure 4(a) presents the electronic absorption spectra of D_2d_ B_40_, the strongest absorption peak occurs at 397 nm and the largest excitation wavelength is 535 nm. Figure 4(b) presents the electronic absorption spectra of quasi-planar B_40_, the strongest absorption peak occurs at 433 nm and the largest excitation wavelength is 1215 nm. Figure 4(a,b) indicate that electronic absorption spectra of quasi-planar B_40_ are apparently red-shifted comparing with B_40_ cage. Figure 4(c) presents the electronic absorption spectra of the C_3_ B_39_^−1^. The computed results show that the two enantiomers have the same electronic absorption spectra. The strongest absorption peak occurs at 476 nm and the largest excitation wavelength is 618 nm. Figure 4(d) presents the electronic absorption spectra of the C_2_ B_39_^−1^, the two enantiomers also have the same electronic absorption spectra. The strongest absorption peak occurs at 399 nm and the largest excitation wavelength is 666 nm. Figure 4(e) presents the electronic absorption spectra of the D_2h_ B_38_ cage. The strongest absorption peak occurs at 437 nm amd the largest excitation wavelength is 949 nm. Note that the oscillator strength of this largest excitation wavelength is zero, and the largest excitation wavelength (with nonzero oscillator strength) is 812 nm. Figure 4(f) presents the electronic absorption spectra of the quasi-planar B_38_. The strongest absorption peak occurs at 434 nm and the largest excitation wavelength is 2588 nm. Similar to B_40_, Fig. 4(e,f) indicate that electronic absorption spectra of quasi-planar B_38_ are apparently red-shifted comparing with B_38_ cage.

Figure 4 indicates that the largest excitation wavelengths of B_40_ (C_s_) and B_38_ are in the near infrared region. One can observe several near infrared (NIR) absorption peaks of the quasi-planar B_40_ and B_38_. The B_40_ (D_2d_) and B_39_^−1^ have only UV-vis spectra. The electronic absorption spectra may be used for the structural analysis in conjunction with other techniques. In addition, Uv-vis spectroscopy can be used to distinguish isomers, such as quasi-planar and cage-like B_40_ with obvious different absorption peaks. The minimum excitation energy (the largest excitation wavelength) mainly comes from the electron transition from HOMO to LUMO. HOMO–LUMO energy gap reflects the probability of the molecules jumping from ground state to excited state. Generally speaking, the larger energy gap can lead to the larger electron excitation energy, i.e., the smaller the probability of electronic transition. On the contrary, the molecule with smaller energy gap is easier to jump to the excited state. According to the previous results, the HOMO–LUMO energy gaps are 3.13, 2.89, 2.73, 2.3, 1.82, 1.33 eV for B_40_ (D_2d_), B_39_^−1^(C_3_), B_39_^−1^(C_2_), B_38_ (D_2h_), B_40_ (C_s_), B_38_ (C_1_), respectively. Although the energy gap of ground state does not represent the minimum excitation energy, the decreasing HOMO–LUMO energy gaps just reflect the increasing largest excitation wavelength 535 nm, 618 nm, 666 nm, 949 nm, 1215 nm, and 2588 nm for B_40_ (D_2d_), B_39_^−1^ (C_3_), B_39_^−1^ (C_2_), B_38_ (D_2h_), B_40_ (C_s_), and B_38_ (C_1_), respectively.

The new discovery of all-boron fullerene has provided an important clue for the development of new boron-based materials. In view of the remarkable structure and property, it is possible to have a potential application in energy, environment, optoelectronic materials and pharmaceutical chemistry. Here the infrared spectra, Raman spectra, and electronic absorption spectra of boron clusters B_n_^0/−1^(n = 38–40) were simulated at the level of density functional theory (DFT) and time-dependent density functional theory (TD-DFT) with 6–311 + G* basis set, these spectra display a large variety of shapes and patterns. Comparative calculations show that size, symmetry, and charge state strongly affect the infrared and Raman spectra, which suggests that infrared and Raman spectra may play a key role in identifying these boron clusters. In addition, the calculated electronic absorption spectra indicate that quasi-planar boron clusters have obvious near-IR absorption peaks. These spectral features provide a theoretical basis in the future experimental measurements and confirmations. Our calculated results also provide much insight into the new doped boron clusters (such as MB_n_(M = Li, Na, K, Rb, n = 38–40)) and boron nanotubes.

## Additional Information

**How to cite this article**: Li, S. *et al.* Comparative study on the spectral properties of boron clusters B_n_^0/−1^(n =38–40). *Sci. Rep.* **6**, 25020; doi: 10.1038/srep25020 (2016).

## Supplementary Material

Supplementary Information

## Figures and Tables

**Table 1:** The symmetries, energies (*E*), HOMO–LUMO energy gaps (*E*_g_), dipole moments (μ) and states of boron clusters B_n_^0/−1^(n = 38–40) optimized at PBE0/6–311+G* level.

	Symmetry	*E*/hartree	*E*_H_/hartree	*E*_L_/ hartree	*E*_g_/eV	μ/ Debye	State
B_40_	D_2d_	−992.6713	−0.23517	−0.11997	3.1334	0	^1^A_1_
B_40_	C_s_	−992.6284	−0.22555	−0.15879	1.8159	3.7023	^1^A’
B_40_^−1^	D_2d_	−992.7556	−0.04867^a^	−0.00022^a^	1.3178^a^	0	^2^B_2_
			−0.11828^b^	−0.00833^b^	2.9906^b^		
B_40_^−1^	C_s_	−992.7562	−0.09707^a^	−0.04757^a^	1.3464^a^	5.9518	^2^A’
			−0.11418^b^	−0.05321^b^	1.6584^b^		
B_39_^−1^(1)	C_3_	−967.9327	−0.10434	0.00206	2.8941	2.3903	^1^A
B_39_^−1^(2)	C_3_	−967.9327	−0.10433	0.00206	2.8938	2.3905	^1^A
B_39_^−1^(1)	C_2_	−967.9280	−0.10382	−0.00357	2.7268	1.4565	^1^A
B_39_^−1^(2)	C_2_	−967.9278	−0.10388	−0.00361	2.7273	1.4518	^1^A
B_39_(1)	C_3_	−967.7889	−0.22608^a^	−0.11441^a^	3.0374^a^	2.1636	^2^A
			−0.22469^b^	−0.17755^b^	1.2822^b^		
B_39_(2)	C_3_	−967.7889	−0.22608^a^	−0.11441^a^	3.0374^a^	2.3660	^2^A
			−0.22469^b^	−0.17755^b^	1.2822^b^		
B_39_(1)	C_2_	−967.7886	−0.22615^a^	−0.12141^a^	2.8489^a^	0.5834	^2^A
			−0.23281^b^	−0.17429^b^	1.5917^b^		
B_39_(2)	C_2_	−967.7884	−0.22618^a^	−0.12140^a^	2.8500^a^	0.5784	^2^A
			−0.23287^b^	−0.17427^b^	1.5939^b^		
B_38_	D_2h_	−942.9736	−0.21258	−0.13043	2.2345	0	^1^A_g_
B_38_	C_1_	−942.9599	−0.22368	−0.17482	1.3290	1.7604	^1^A
B_38_^−1^	D_2h_	−943.0672	−0.05699^a^	0.01341^a^	1.9149^a^	0	^2^B_2u_
			−0.08921^b^	−0.01327^b^	2.0656^b^		
B_38_^−1^	C_1_	−943.1114	−0.11565^a^	−0.04141^a^	2.0193^a^	5.1377	^2^A
			−0.11561^b^	−0.04122^b^	2.0234^b^		

The superscripts a and b denote the alpha electron and beta electron, respectively.
