# Supplementary material for: Comparative study on the spectral properties of boron clusters Bn0/−1(n = 38–40)
Source: Sci Rep. 2016 Apr 26;6:25020. doi: 10.1038/srep25020 (PMC4844962; doi:10.1038/srep25020)
Supplement: Supplementary Information [file srep25020-s1.doc]

**Comparative study on the spectral** **properties of boron clusters B_n_^0/-1^(n=38-40)**

Shixiong Li ^1, 2^, Zhengping Zhang ^1,*^, Zhengwen Long ^3^, Guangyu Sun ^2^, and Shuijie Qin ^4^

1 College of Big Data and Information Engineering, Guizhou University, Guiyang 550025, China

2 School of Physics and Electronic Science, Guizhou Education University, Guiyang 550018, China

3 College of Science, Guizhou University, Guiyang 550025, China

4 Key Lab of Photoelectron Technology and Application, Guizhou University, Guiyang 550025, China

email: [zpzhang@gzu.edu.cn](mailto:zpzhang@gzu.edu.cn)

email: [leesxoptics@163.com](mailto:leesxoptics@163.com)

**Figure S1**. Optimized Structures of the boron clusters B_n_^0/-1^ (n=38-40) at the PBE0/6-311+G* level.


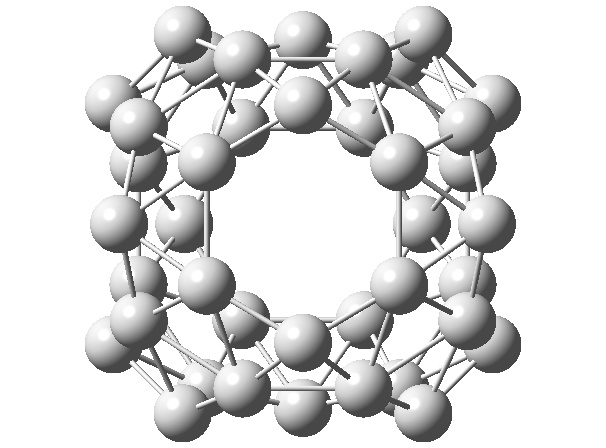

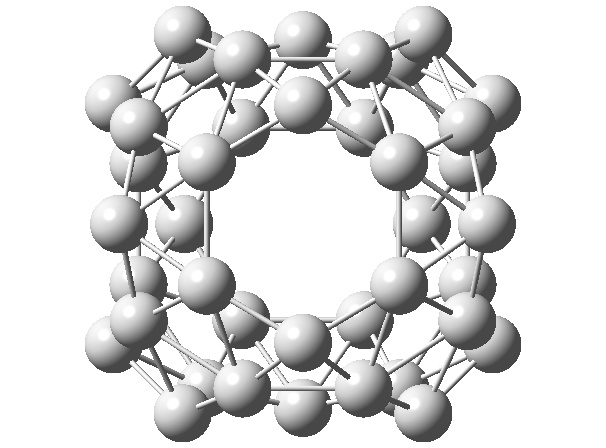


(a) D_2d_ B_40_ (^1^A_1_) (b) D_2d_ B_40_^-1^ (^2^B_2_)


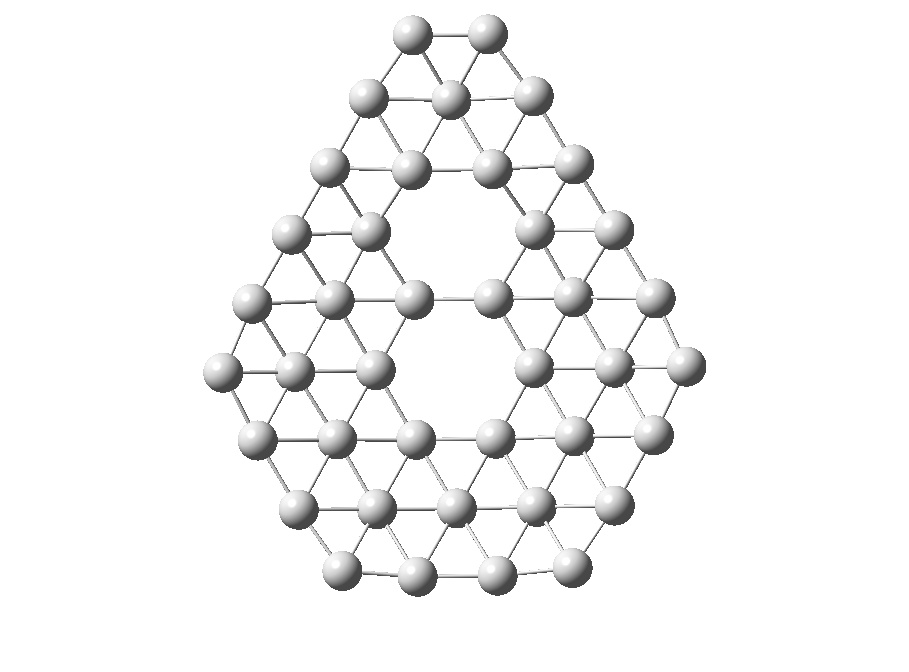

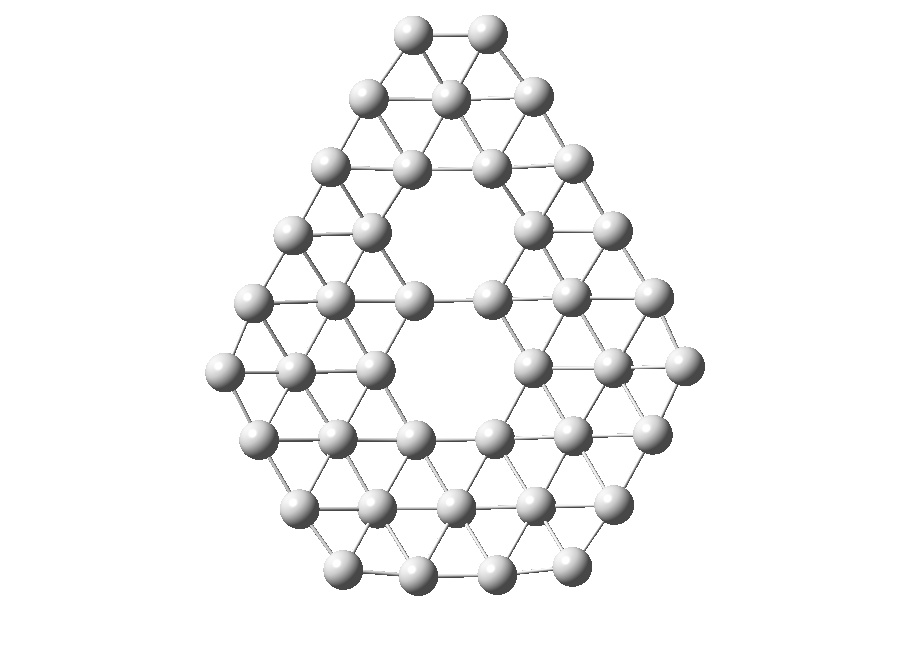


(c) C_s_ B_40_ (^1^A’) (d) C_s_ B_40_^-1^ (^1^A’)


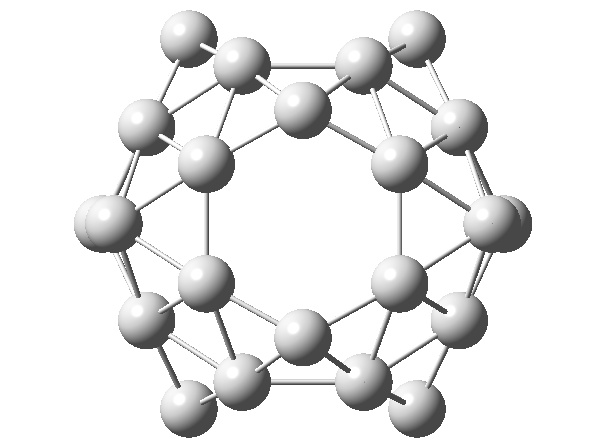

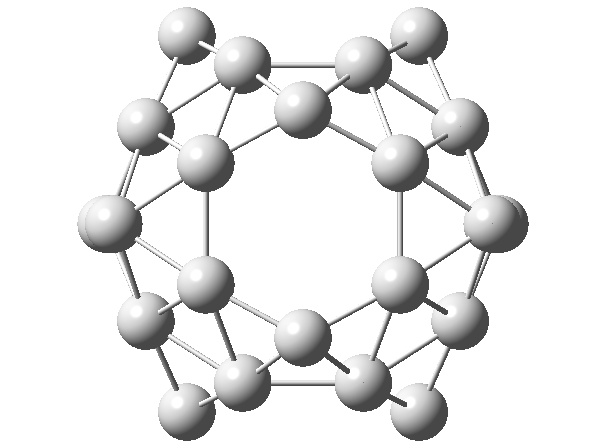


(e) D_2h_ B_38_ (^1^A_g_) (f) D_2h_ B_38_^-1^ (^2^B_2u_)


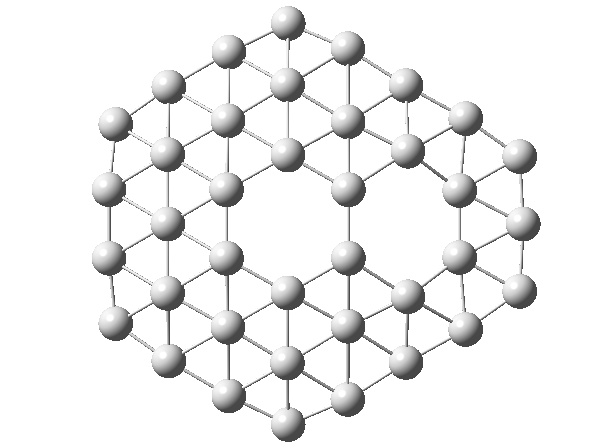

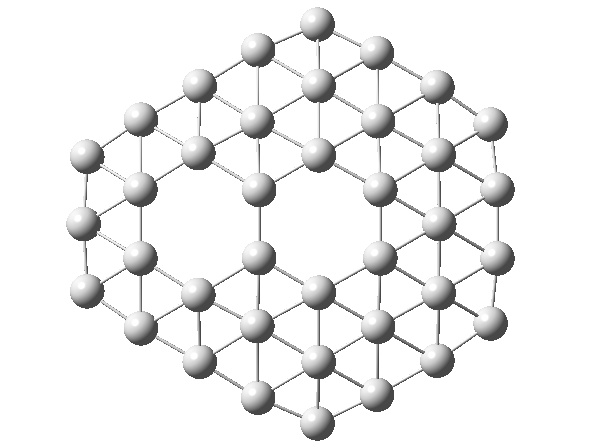


(g) C_1_ B_38_ (^1^A) (h) C_1_ B_38_^-1^ (^2^A)


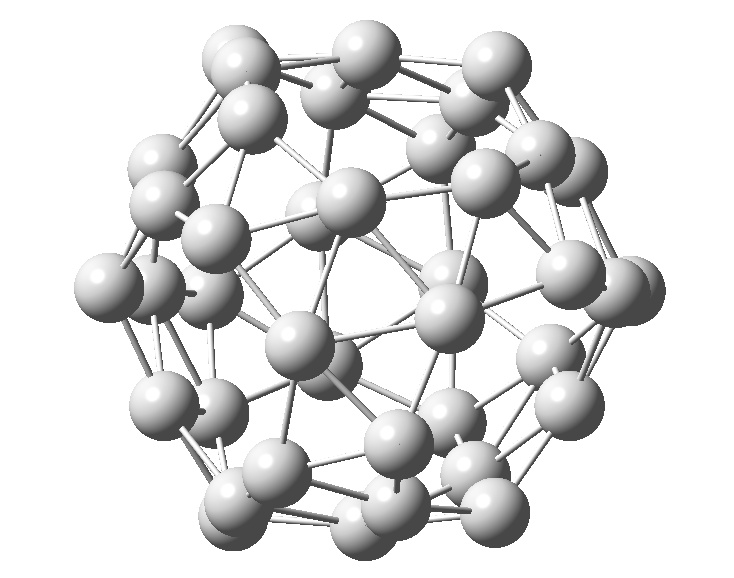

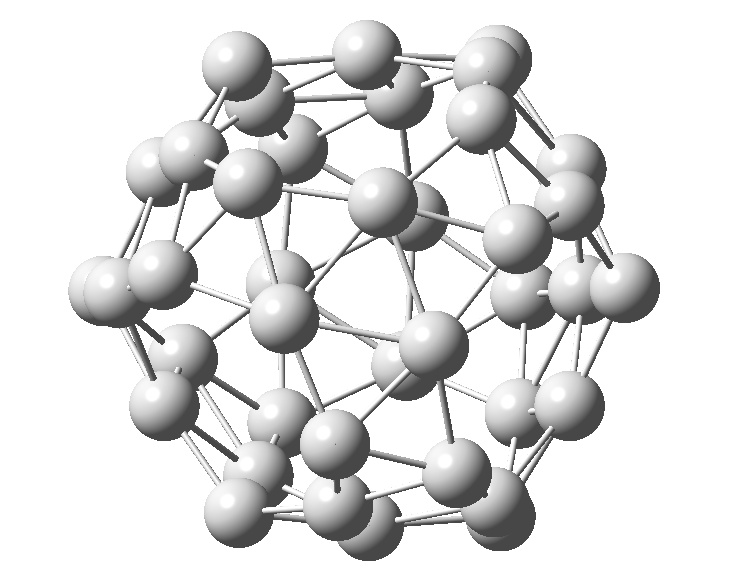


(i) C_3_ (1) B_39_^-1^ (^1^A) (j) C_3_ (2) B_39_^-1^ (^1^A)


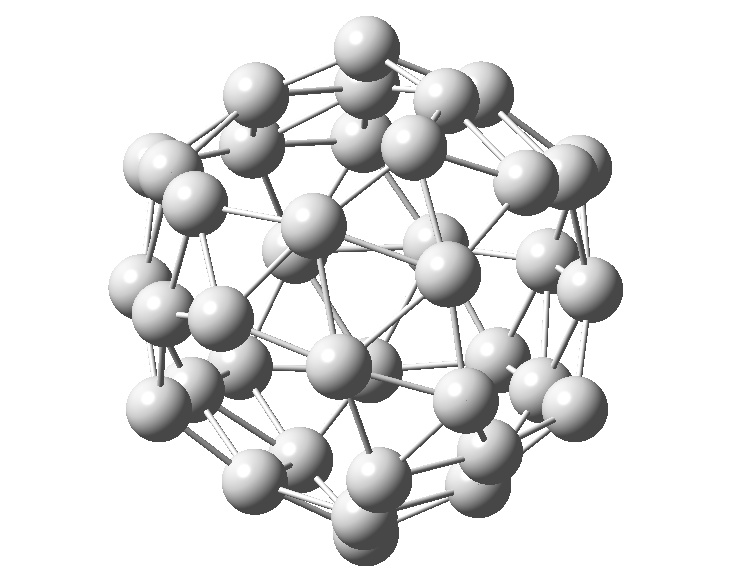

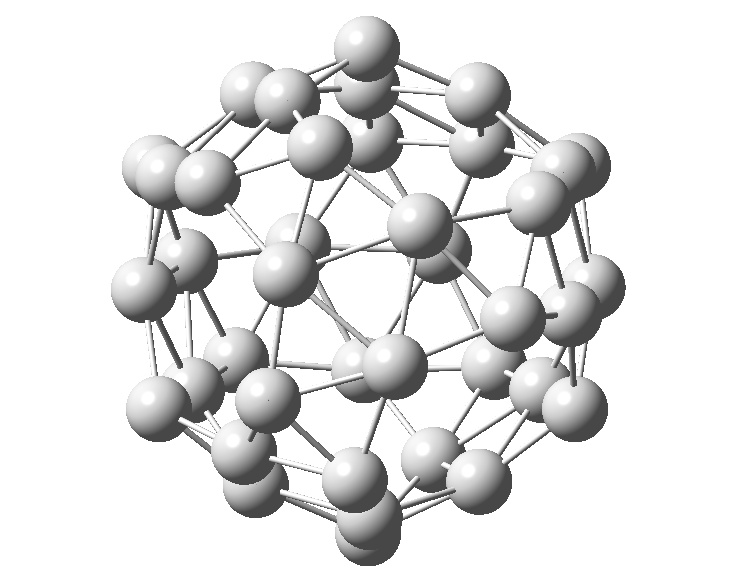


(k) C3 (1) B_39_ (^2^A) (l) C3 (2) B_39_ (^2^A)


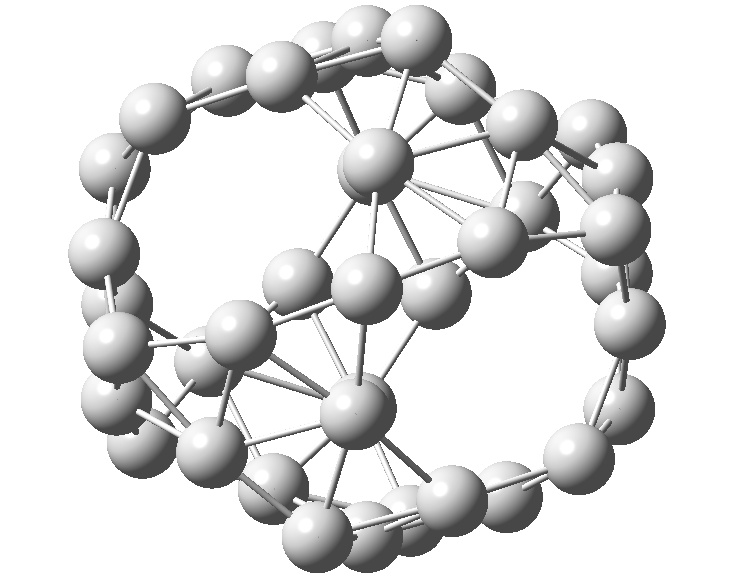

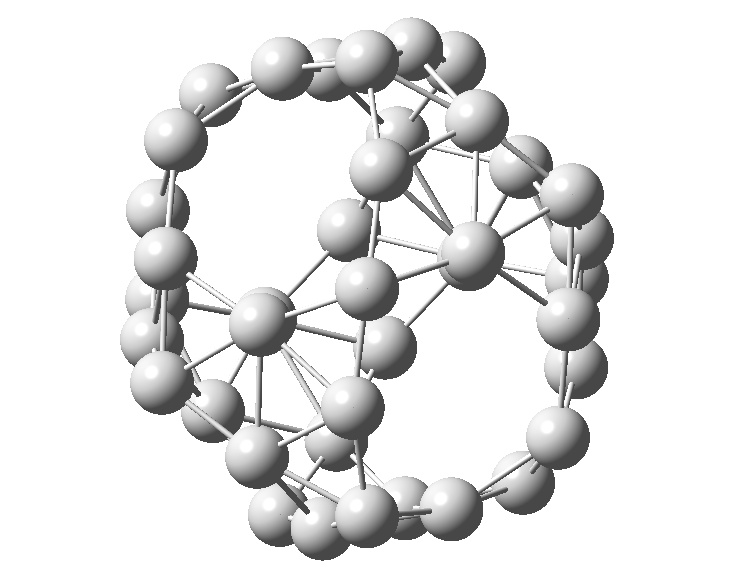


(m) C2 (1) B_39_^-1^ (^1^A) (n) C2 (2) B_39_^-1^ (^1^A)


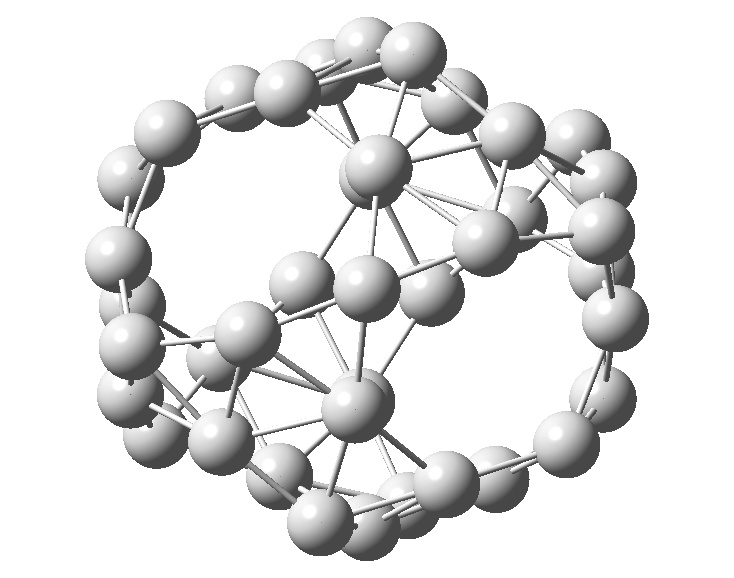

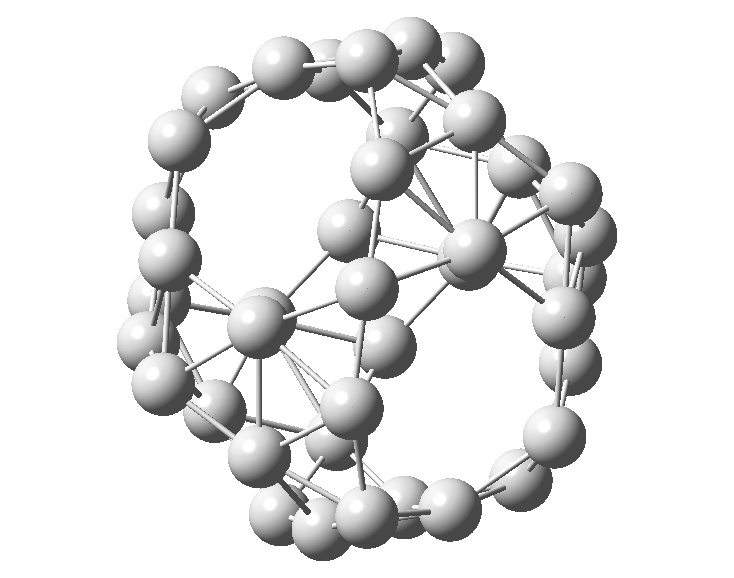


(o) C2 (1) B_39_ (^2^A) (p) C2 (2) B_39_ (^2^A)
